# Air Pollution and Adolescent Development: Evidence from a 3-Year Longitudinal Study in China

**DOI:** 10.3390/children8110987

**Published:** 2021-11-01

**Authors:** Na Ni, Xinli Chi, Wei Liu, Xiumin Cui

**Affiliations:** 1 Webank Institute of Fintech, Shenzhen Audencia Business School, Shenzhen University, Shenzhen 518060, China; 2 School of Psychology, Shenzhen University, Shenzhen 518060, China; xinlichi@szu.edu.cn; 3 School of Management, Xiamen University, Xiamen 361005, China; liuwei917@stu.xmu.edu.cn; 4 College of Education for the Future, Beijing Normal University, Zhuhai 519087, China; cuixiumin13@126.com; 5 The Affiliated Kindergarten of Meilian Primary School, Shenzhen 518035, China

**Keywords:** adolescent development, air quality, emotional disorders, China, descriptive survey study

## Abstract

This study aimed to investigate the impact of air pollution on the development of adolescents and the mediating role of students’ emotional disorders. Participants came from a longitudinal sample group of adolescents (*n* = 1301) in Southern China from the years 2016 to 2018. They were assessed for the Positive Youth Development and emotional disorders, and air pollution was measured by the Air Quality Index. The results show that students’ higher degree of exposure to air pollution was negatively associated with their positive development. Three out of four emotional disorders (i.e., anxiety, neuroticism, and withdrawal) mediate this association. The results suggest that the physical environment can have a paramount influence on the emotional status and overall development of adolescents, calling for intervention programs by policymakers.

## 1. Introduction

Scholars addressing child and adolescent developmental issues globally have paid substantial attention to the related concepts, antecedents, intervention programs, and consequences [1,2,3,4]. Considering their pervasiveness and negative consequences for personal growth and societal processes, it is vital for various actors, including governments, academia, school educators, and parents, to put more effort into preventing such developmental issues. In particular, researchers need to understand the underlying factors and mechanisms in order to explain different developmental issues among such age groups.

In line with the predictions of ecological system theory [5], the environment can have a paramount influence on the development of adolescents. While the notion of the environment is largely treated as a social condition in the field of psychology, a handful of studies have investigated the physical environment or physical context [6,7], i.e., the “physical properties of the settings and objects people interact with” [8], and argued for its association with individual human development. Air quality, for example, has emerged as a critical physical element, although sometimes as a distal factor of the physical surroundings, that influences the mental health, capacity development, and behavior patterns of adolescents.

Air pollution, a byproduct of the process of industrialization and urbanization in most countries, turns out to be a major cause of the deterioration of human beings’ quality of life. Various statistics show that exposure to toxic air may lead to acute and long-term health problems, such as heart disease, stroke, lung cancer, asthma, and other respiratory issues, for both adults and children [9,10]. It also affects human beings in the form of lowered cognitive performance, defensive or unethical behavior, or other spill-over behaviors [11]. In addition, extant studies in other disciplines, such as economics and business management, focus on the impact of air pollution on individuals’ productivity and investment decision-making [12,13,14,15]. Overall, air pollution has been shown to be a major risk factor for human beings. More recently, some scholars have focused on whether people exposed to high air pollution, including nitrogen dioxide (NO_2_), sulfur dioxide (SO_2_), particulate matter 2.5 (PM_2.5_), ozone (O_3_), etc., suffer from psychological distress [16], greater unhappiness [17,18], anxiety or depression symptoms [17,19,20,21], or other negative mental health issues [22,23]. For example, life dissatisfaction, hedonic unhappiness, and depression are found to be statistically associated with air pollution in multiple cities in China [18]. Therefore, such emotional states are indispensable factors for understanding the role of air pollution on human beings. Nevertheless, most of these studies investigate older adults as their subjects, while studies with a close-up focus on adolescents are still emerging across different institutional contexts.

The studies on the impact of air pollution on adolescent development are also highly focused on physical health outcomes and cognitive development, including children’s hospitalization, learning abilities, school absence, or campus violence [24,25,26,27,28,29]. Similar research focuses exist in studies using a sample of Chinese students [30,31]. Nevertheless, these related studies examined children’s school attainment or test scores as the outcome variables, while paying insufficient attention to how air pollution affects their emotional state and, eventually, their development (i.e., behavioral or capacity outcomes) in a full picture.

By applying existing knowledge about environmental psychology [32], we aim to fill this research gap, and argue that emotional state is the underlying mechanism that explains the impact of air quality on adolescent development. We investigate this association in a longitudinal sample group comprising adolescents in grades seven to nine in Shenzhen, China, from the years 2016 to 2018. In particular, our research question investigates the impact of air pollution, a reliably recorded environmental stressor, on the emotional status and development of adolescents. To further delimitate the specific mechanisms to explain such associations, we use several key dimensions of emotional disorders (e.g., anxiety, neuroticism, withdrawal motivations, etc.) that are particularly related to adolescent development [33,34], and find them related to behavioral changes as the main mediation mechanism. These effects hold after we control for major confounding factors at different levels.

By exploring the effects of air pollution on the same student cohort in terms of their emotional development, as well as behavioral and cognitive outcomes, we aim to contribute to the theory and practice in several aspects. First, we contribute to the extant research in the field of environmental psychology, as we can precisely identify both the relationship between air quality and adolescent’s development and the triggering mechanisms of emotional disorders. Indeed, an in-depth understanding of adolescent development should encompass elements including emotions, internal attributes, behavior patterns, and capacity-building. Second, unlike the major studies in this area [17], this paper uses a unique longitudinal design that tracks changes and associations between air quality, emotional status, and development of the same group of adolescents. Without a longitudinal study to identify the continuous relationship among the variables, our suspicions of a relationship between air quality and child development would remain inconclusive and underdeveloped, and incapable of providing any normative suggestions to practitioners. Third, in practice, our research question is both relevant and timely, as adolescent development is key for society’s human capital attainment, and this is particularly true for developing countries in Asia, where sophisticated prevention and intervention systems are still lacking [1]. By identifying the relationship between air quality, emotional status, and adolescent development, this longitudinal study hopes to move forward the related policy making process.

## 2. Methods

### 2.1. Participants

The reported study was based on multiple waves of data collected during three junior adolescent years in Shenzhen, China. We initially approached six schools, which were randomly selected from the nine districts of Shenzhen, and one school in each district was invited to participate in the study. At Wave 1 (October 2016), 1544 adolescents in the 7th grade participated in the study. The data collection at Wave 2 was one year later (October 2017), and 1511 adolescents filled in the questionnaire, all of whom had participated at Wave 1 (and non-participation was mainly due to student dropout). At Wave 3 (October 2018), the data were collected when these students were in the 9th grade, with 1480 of them completing the survey. Finally, a total of 1301 adolescents provided data for all three waves, which included 666 males and 621 females, and 14 students did not report their gender. The mean age of participants was 12.46 years (SD = 0.63) at Wave 1. To address the missing data issue, we apply random interpolation for category variables, and replace with mean value for continuous variables. A final of 3903 observations are used in our study.

### 2.2. Procedure

The survey was carried out in the participants’ schools. Consent was obtained from the school, parents, and respondents beforehand. Each participant was assigned a unique ID that was used for all three waves. The students were invited to fill in a paper-based questionnaire that included scales of main variables. The study and data collection were approved by the administration committees of the surveyed schools and the human research ethics committee of the affiliated university (No. 20160516, approved on 22 June 2016).

### 2.3. Measurements

#### 2.3.1. Positive Youth Development Scale

We used the Positive Youth Development scale (PYD) [35] to investigate participants’ positive youth development in China. We dropped the three items related to students’ spirituality from the original scale as they are not applicable to adolescents in China. Therefore, the PYD used in this study consists of 41 items, divided into four second-order dimensions that reflect adolescents’ cognitive behavioral competence (CBC), prosocial attributes (PA), positive and healthy identity (PIT), and general positive youth development qualities (GPYDQ). Each item was rated on a six-point Likert scale. Global positive youth development was reflected by the mean scores of the 44 items, and four second-order factor scores were calculated by averaging the scores of related items. The PYD has been widely used in previous studies with good reliability and validity [35]. In this study, the PYD showed acceptable reliability and validity (see Table 1).

#### 2.3.2. Internalizing Disorders Scale

‘Internalizing disorders’ in this study refers to emotional problems or felt distress in individuals, and was used to assess Chinese adolescents [33] with 21 items that collectively measure anxiety (6 items, e.g., I’m always nervous and hard to calm down), depression symptoms (9 items, e.g., I feel unhappy and miserable), neuroticism (3 items, e.g., I am worried that I will suddenly encounter misfortune), and withdrawal motivation (3 items, e.g., I am stupid and unwilling to attend school). All items were rated on a five-point Likert scale. Higher total scores represent more internalizing problem behaviors. The scale showed high internal consistency and good fit indices in confirmatory factor analysis (see Table 2).

#### 2.3.3. Socio-Demographic Characteristics

Participants’ sociodemographic characteristics were measured separately with a single item, including gender, place of growth, only child or not, and migrant or local resident status. In addition, they responded to questions about their parents’ educational levels, per capita monthly household income, and family intactness.

#### 2.3.4. Independent Variables—Air Pollution

The air pollution data was derived from the national real-time air quality database released by the CNEMC (China National Environmental Monitoring Center, http://www.cnemc.cn/, accessed on 20 November 2020). The CNEMC primarily adopts two sets of standards, the “Ambient Air Quality Standards” (GB3095-2012) and “Technical Regulation on Ambient Air Quality Index (on trial)” (HJ633-2012), and mainly releases data on air quality from more than 1400 environmental monitoring stations in China implemented from 2016. It releases the most authoritative data to the general public that measures air quality across cities in China [36]. We use Stata15.1 to process and analyze the data.

We measure the degree of air pollution by using the Air Quality Index (AQI), a quantitative description that the public and government generally pay attention to [18,30,37]. The value range of China’s AQI is similar to that of the United States, ranging from 0 to 500. The larger the value, the more severe the air pollution. AQI is constructed based on the levels of six atmospheric pollutants: sulfur dioxide (SO_2_), nitrogen dioxide (NO_2_), suspended particulates smaller than 10 μm in aerodynamic diameter (PM_10_), suspended particulates smaller than 2.5 μm in aerodynamic diameter (PM_2.5_), carbon monoxide (CO), and ozone (O_3_), which gives a comprehensive reflection of the air quality in the local geographic area.

**Figure 1:**
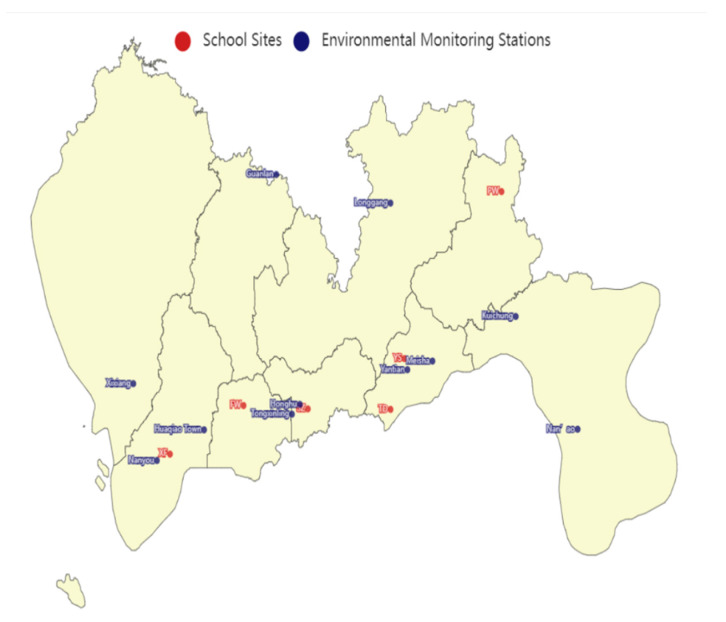
The distribution of environmental monitoring stations and schools.

In order to accurately measure the school-level AQI, we matched each school to the closest monitoring site based on the school’s geographic location (see Figure 1), and used this as an initial indicator to measure the school’s AQI. Following the data processing methods in previous studies, the initial indicators were centrally processed, denoted as $AQI_{kt}$. As existing literature in the medical field shows that the impact of air pollution on people has a lagged effect [33], the variables affected by air quality were treated as a lag.

### 2.4. Statistical Model

We used the multiple regression model and ordinary least squares (OLS) to test the impact of air pollution on the developmental status (*PYD*) of adolescents. Our main analyses are based on specifications of the following form:

(1) $$PYD_{it}=\alpha +\beta \times AQI\_School_{kt}+\gamma \times X_{it}+\epsilon_{it}$$

As shown in Model 1, *PYD_it_* represents the *PYD* score of Student *i* in Year *t*, $AQI\_School_{kt}$ represents the *AQI* of School *k* in Year *t*, and *X_it_* represents control variables at different levels that may have an impact on *PYD*. In this part, we reduced all technical variables by one, and the continuous variable data were processed centrally. According to our expectations, we predicted that β is significant.

## 3. Results

### 3.1. Descriptive Analysis

Table 3 shows the descriptive statistics of this study. We included the tracking data of 1301 students for three years in this study, which are classified into categorical and continuous variables, respectively. For categorical variables, boys and girls account for 51.65% and 48.35% of the whole sample, respectively. Among them, 61.54% of the students are not only children. Most students are local, accounting for 81.66%. In addition, 74.51% of the students grew up in Shenzhen. As for their family background, fathers and mothers who have a bachelor’s degree or higher account for 30.11% and 25.62%, respectively. Most of the students in our sample are growing up in complete families, accounting for 94.36% of the total. The per capita household income is relatively uniform: 36.41% earn over RMB six thousand (about US$950) per month.

As for the two continuous variables, the average value of PYD (PYD_Total) score is 4.872. Its minimum (maximum) value is 1 (6), and the variance is 0.738. In addition, the average AQI of each school (AQI_School) is 38.65, ranging between 33.94 and 47.41. According to the AQI level, the air quality in the area is at a relatively good level.

### 3.2. Main Effect

We present the multiple regression results of model (1) in Table 4. In column 1, it can be seen that the regression coefficient between the air quality index (AQI_School) and the youth PYD score is −0.017 (*t* value = −4.94) and is significant at the 1% level. It indicates that the worse the air quality, the lower the PYD score. Similar associations exist for the four sub-dimensions of PYD (i.e., cognitive behavioral competence, prosocial attributes, positive and healthy identity, and general positive youth development qualities) in columns two to five. Overall, air pollution has a negative impact on the growth of adolescents. For the results of the control variables, the coefficients of student gender and family intactness on PYD score are significantly negative, and those of parents’ educational level and family income are significantly positive.

### 3.3. Mediation Effects

We further explored the internal mechanism of air pollution on the PYD of surveyed students by using the overall internalizing disorders score and the four dimensions individually (i.e., anxiety, depression, neuroticism, and withdrawal) to analyze the mechanisms. Referring to prior research, we use the mediation effect model for empirical testing. Table 5 and Table 6 show the regression results in a two-step analysis.

In step one, Table 5 shows that the regression coefficients of the air pollution index on the total internalizing disorders, anxiety, neuroticism, and withdrawal behaviors are 0.118, 0.042, 0.028, and 0.021. They are all significant at the 10% or 5% level, indicating that the more serious the air pollution, the stronger the internalizing disorders either in total or among these three sub-dimensions. Interestingly, although the regression coefficient of AQI_School in the third column of Table 5 is positive, it is not significant. This indicates that the impact of air pollution on adolescent depression is not very clear.

In step two, Table 6 shows the results when the four main variables that passed step one in testing mediation effects (i.e., total internalizing disorders, anxiety, neuroticism, and withdrawal behaviors) and explanatory variables are introduced into the model at the same time. The coefficient of AQI_School is still significantly negative as expected, but the absolute value of the coefficient was slightly lower than that of the main effect (Table 4). Furthermore, the coefficients of these four types of emotion variables are all significantly negative, indicating their mediating roles. That is, air pollution increases adolescents’ overall emotional disorders, as well as the sub-dimensions of anxiety, neuroticism, and withdrawal motivation, and ultimately leads to a decrease in the development of adolescents.

## 4. Discussion

Our research findings are largely consistent with previous findings of the negative impact of air pollution on children’s development. By adding to the literature on whether air pollution affects adolescents’ physical health status, cognitive performance, and school attendance [17,29,30], with this study we have made our first theoretical contribution by focusing on the impact of the physical environment, and specifically air quality, on a much broader concept of youth development, the PYD. We found that exposure to more severe air pollution was associated with lower PYD. The results were robust with the analyses conducted for both the aggregated PYD measure and individual ones. Therefore, it provides substantial support for the notion that air pollution is a substantial risk factor for the development of adolescents. In particular, adolescent development should be perceived as a total concept, incorporating elements regarding adolescents’ behavioral competence, identity, prosocial attributes, and other general qualities. In addition, our robustness check shows that the impact of air quality on adolescent development holds for the whole time period of three years, and for each individual year. Therefore, toxic air is obviously a fundamental threat to the growth of adolescents.

Our second theoretical contribution shows the specific mechanisms of how air pollution eventually makes adolescents vulnerable to such an adverse problem; therefore, it provides a complete framework to understand the exact paths that relate air pollution to adolescent development. Furthermore, the longitudinal design of this study with lagged effects of air quality on emotions and development reminds us of the extended influence of air quality. Overall, the empirical findings verify that school children are more likely to suffer emotional disorders due to exposure to air pollution, which further leads to deteriorated development. For example, we demonstrate that air quality may trigger emotional disorders in students, such as symptoms of anxiety, neuroticism, and withdrawal motivations, which further leads to the low PYD. This is largely consistent with the current literature [19,20,21]. We posit that anxiety is a natural reaction to toxic levels of pollution, which may make adolescents unsatisfied with the current situation, lower their subjective well-being, and make it difficult for them to sustain their personal development potential. 

Interestingly, the current empirical findings do not show a positive relationship between air pollution and depression in our participants. This may be because they are adolescents. Usually, depressive symptoms as a result of adverse air quality are more prevalent in the elderly, as shown in previous studies [23,38]. In contrast, the emotional disorders of adolescents who are at the early stage of their lives are more likely to be reflected as anxiety toward the future, social isolation, or withdrawal intentions from others, as well as neuroticism with unstable moods. Indeed, it is particularly valuable for academia and policymakers to investigate the negative effects of air pollution on an array of populations, and develop corresponding prevention mechanisms effectively.

It should be noted that China is a less aged country, with one fifth of the population consisting of children who are less than 18 years old. Undoubtedly, such a negative impact of low air quality may lead to major social costs for adolescent development resulting from high expenditures in the public health service and undermined human capital development for the country. For example, it was estimated that air pollution leads to annual economic losses that amount to 1% to 3.8% of GDP, considering the two factors of direct medical costs and willingness to pay [39]. It will eventually cause more personal suffering and economic problems that are a substantial burden to the society on a global level. Essentially, the burgeoning industrialization process over the past forty years in China is as double-edged sword; that is, both economic development and ecological degradation are intertwined, which requires urgent solutions.

We recognize that our future research can be improved in several aspects. First, the findings may be constrained by the single research site of Shenzhen, which is often perceived as one of the cleanest cities in China. Nevertheless, given the uneven distribution of environmental conditions in China, the overall interpretation should be cautious as the adverse association between air quality and students’ emotional health and development may vary in other areas. A comparative study including adolescents from other places or countries is warranted [40]. Second, while our current focus is the composite measure of air pollution, AQI, additional analyses of other indicators are required to show if there are any individual effects on adolescents that are hidden by our combined effects. In addition, we also need to control for potential environmental confounders, such as daily temperature, relative humidity, and duration of sunlight. Future research needs to assess such variance across different pollutants and health outcomes. Fourth, our results must be interpreted with caution, as there are always confounding factors related to potential residual and unmeasured errors.

This study has several theoretical and practical implications. First, our research question is highly relevant, as ambient air pollution has become one of the major environmental hazards to health in the last decades, including damage to the brain and respiratory system, cognitive performance, and with cardiovascular effects [38,41,42,43]. Therefore, we aim to bring the attention of academic researchers and related parties in the adolescent development setting (i.e., children, parents, and schools) in order to prevent and minimize such damage. Secondly, government, as policymakers, needs to play an important role in advancing air quality governance and taking effective actions to combat it. They need to fully understand whether there is a causal impact of air pollution on human health issues, as found in the current study. In addition, as air quality may further affect individuals’ participation in economic activities, productivity, and the future economic development of nations, policy makers need to formulate numerous policies and incentive schemas to curb different sources of air pollution. Third, players in the private sector and non-profits can also take collaborative efforts and focus on how to leverage resources from multiple sources to prevent the occurrence of more air pollution, which will benefit adolescent development in the long run.

## 5. Conclusions

For adolescents whose immune systems are still in the developmental stage, spending more time outside with exposure to degraded, toxic air makes them more vulnerable. By paying particular attention to the impact of air pollution on the emotional health and capacity-building of adolescents, such as positive youth development programs in this study, we hope to catch the attention of the local governments to invest more in air pollution schemas and practices in order to minimize the associated risks on adolescents. We hope to engender a better understanding of the main factors and lead to evidence-based policymaking for primary prevention, and for specific interventions from policy-makers and other related parties in society.

## Figures and Tables

**Table 1:** Psychometric characteristics of PYD.

Scales	Χ^2^	*df*	CFI	TLI	RMSEA	SRMR	Cronbach’s α
CBC-W1	4141.522	36	0.992	0.975	0.046	0.015	0.861
CBC-W2	4582.095	36	0.997	0.989	0.032	0.012	0.871
CBC-W3	8326.689	36	0.995	0.986	0.049	0.010	0.932
PA-W1	1882.882	10	0.999	0.995	0.026	0.007	0.796
PA-W2	2110.620	15	0.998	0.993	0.028	0.007	0.780
PA-W3	5441.852	15	0.996	0.978	0.078	0.014	0.906
PIT-W1	3612.898	15	0.995	0.983	0.056	0.000	0.870
PIT-W2	3872.282	15	0.992	0.970	0.077	0.014	0.881
PIT-W3	6005.443	15	0.999	0.997	0.030	0.004	0.922
GPYDQ-W1	10,425.744	190	0.933	0.917	0.059	0.042	0.913
GPYDQ-W2	11,772.895	190	0.935	0.916	0.063	0.043	0.921
GPYDQ-W3	19,814.318	190	0.968	0.959	0.057	0.029	0.957

Note. W1 = Wave 1 (first year); W2 = Wave 2 (second year), W3 = Wave 3 (third year). CBC: cognitive-behavioral competencies; PA: prosocial attributes; PIT: positive identity; GPYDQ: general positive youth development qualities.

**Table 2:** Psychometric characteristics of Internalizing Disorders.

Scales	Χ^2^	*df*	CFI	TLI	RMSEA	SRMR	Cronbach’s α
Anxiety-W1	2038.381	15	0.978	0.963	0.062	0.023	0.802
Anxiety-W2	2330.065	15	0.969	0.948	0.079	0.027	0.818
Anxiety-W3	3363.749	15	0.982	0.966	0.076	0.022	0.868
Depression-W1	3847.853	36	0.959	0.941	0.069	0.034	0.852
Depression-W2	4506.363	36	0.966	0.951	0.068	0.030	0.870
Depression-W3	6868.074	36	0.980	0.970	0.066	0.024	0.902
Nervousness-W1	957.858	3	1.000	1.000	0.000	0.000	0.756
Nervousness-W2	850.984	3	1.000	1.000	0.000	0.000	0.737
Nervousness-W3	1221.998	3	1.000	1.000	0.000	0.000	0.797
Withdrawal motivation-W1	519.411	3	1.000	1.000	0.000	0.000	0.520
Withdrawal motivation-W2	689.193	3	1.000	1.000	0.000	0.000	0.584
Withdrawal motivation-W3	722.762	3	1.000	1.000	0.000	0.000	0.675

Note. W1 = Wave 1 (first year); W2 = Wave 2 (second year), W3 = Wave 3 (third year).

**Table 3:** Descriptive statistical analysis.

Categorical Variables	*N*	%		*N*	%
Gender			Mother_Degree		
Male	2016	51.65	Junior high and below	1537	39.38
Female	1887	48.35	High school or junior college	1366	35.00
One_Child			Bachelor	715	18.32
Yes	1501	38.46	Master’s degree or above	285	7.30
No	2402	61.54	Family		
Local			Full	3683	94.36
Yes	3187	81.66	Parental divorce	124	3.18
No	716	18.34	Single parent family	72	1.84
Growth_Place			Other	24	0.61
Country	475	12.17	Income (family per-capita, in RMB)		
Shenzhen	2908	74.51	Below 1000	163	4.18
Other Cities	520	13.32	1000~1999	332	8.51
Father_Degree			2000~2999	493	12.63
Junior high and below	1298	33.26	3000~3999	577	14.78
High school or junior college	1430	36.64	4000~4999	447	11.45
Bachelor	747	19.14	5000~5999	470	12.04
Master’s degree or above	428	10.97	Above 6000	1421	36.41
Continuous variables	*N*	mean	s.d.	p50	range
PYD_Total	3903	4.872	0.738	4.929	1~6
AQI_School	3903	38.65	3.887	37.864	33.942~47.407

Note. All categorical variables are then transformed with numerical values when entering into the regression model, which include Gender (0 = male, 1 = female), One_Child (0 = only child, 1 = has siblings), Local (0 = migrant, 1 = local resident), Growth_Place (0 = village, 1 = Shenzhen, 2 = other cities), Father_Degree (0 = less than or middle school, 1 = high school and college, 2 = university, and 3 = more than university), Mother_Degree (0 = less than or middle school, 1 = high school and college, 2 = university, and 3 = more than university), Income (0 < 1000, 1 = between 1000 and 1999, 2 = between 2000 and 2999, 3 = between 3000 and 3999, 4 = between 4000 and 4999, 5 = between 5000 and 5999, and 6 =≥ 6000), and Family (0 = intact, 0 = non-intact).

**Table 4:** The results of multiple regression analysis.

	(1)	(2)	(3)	(4)	(5)
	PYD_Toal	CBC	PA	PIT	GPYDQ
AQI_School	−0.017 ***	−0.016 ***	−0.007 *	−0.009 **	−0.016 ***
	(−4.94)	(−4.24)	(−1.73)	(−2.03)	(−4.53)
Gender	−0.057 **	−0.123 ***	0.044	−0.170 ***	−0.02
	(−2.43)	(−4.69)	(1.58)	(−5.49)	(−0.82)
Growth_Place	0.024	0.04	0.011	0.049	0.01
	(0.99)	(1.52)	(0.38)	(1.59)	(0.41)
One_Child	−0.021	−0.033	0.011	−0.053	−0.009
	(−0.80)	(−1.13)	(0.37)	(−1.55)	(−0.35)
Local	0.047	0.042	0.048	0.039	0.047
	(1.52)	(1.22)	(1.33)	(0.97)	(1.51)
Father_Degree	0.054 ***	0.056 ***	0.035 *	0.068 ***	0.049 ***
	(3.32)	(3.10)	(1.84)	(3.14)	(2.91)
Mother_Degree	0.068 ***	0.068 ***	0.073 ***	0.074 ***	0.060 ***
	(3.96)	(3.58)	(3.64)	(3.29)	(3.46)
Family	−0.169 ***	−0.075	−0.229 ***	−0.301 ***	−0.166 ***
	(−3.31)	(−1.32)	(−3.82)	(−4.51)	(−3.19)
Income	0.018 ***	0.022 ***	0.007	0.015 *	0.019 ***
	(2.75)	(3.12)	(0.97)	(1.76)	(2.85)
_Cons	5.335 ***	5.283 ***	5.069 ***	5.039 ***	5.352 ***
	(39.92)	(35.67)	(32.21)	(28.79)	(39.29)
*N*	3903	3903	3903	3903	3903
Adj-R^2^	0.025	0.025	0.014	0.03	0.019

Notes. ***, **, * are significant at the 1%, 5%, and 10% levels, respectively. The *t*-value is in parentheses.

**Table 5:** The tests of mediating mechanisms: Step 1.

	(1)	(2)	(3)	(4)	(5)
	Internalizing Disorders	Anxiety	Depression	Neuroticism	Withdrawal
AQI_School	0.118 *	0.042 *	0.027	0.028 **	0.021 **
	(1.70)	(1.81)	(0.85)	(2.21)	(2.10)
Gender	2.530 ***	1.164 ***	1.127 ***	0.151 *	0.089
	(5.41)	(7.50)	(5.22)	(1.75)	(1.35)
Growth_Place	−0.271	−0.03	−0.147	−0.037	−0.057
	(−0.57)	(−0.19)	(−0.68)	(−0.43)	(−0.86)
One_Child	0.125	−0.055	0.031	0.052	0.097
	(0.24)	(−0.32)	(0.13)	(0.54)	(1.31)
Local	−0.731	−0.214	−0.419	−0.061	−0.037
	(−1.20)	(−1.06)	(−1.49)	(−0.55)	(−0.42)
Father_Degree	−1.230 ***	−0.404 ***	−0.480 ***	−0.139 **	−0.206 ***
	(−3.78)	(−3.75)	(−3.20)	(−2.32)	(−4.47)
Mother_Degree	−0.631 *	−0.189 *	−0.318 **	−0.086	−0.038
	(−1.87)	(−1.69)	(−2.04)	(−1.38)	(−0.80)
Family	4.386 ***	1.222 ***	2.133 ***	0.556 ***	0.474 ***
	(4.34)	(3.65)	(4.58)	(2.99)	(3.31)
Income	−0.199	−0.046	−0.072	−0.044 *	−0.037 **
	(−1.57)	(−1.10)	(−1.23)	(−1.88)	(−2.06)
_cons	35.957 ***	10.623 ***	16.342 ***	4.683 ***	4.309 ***
	(13.58)	(12.10)	(13.38)	(9.60)	(11.46)
*N*	3903	3903	3903	3903	3903
adj. *R*^2^	0.023	0.025	0.022	0.008	0.016

Notes. ***, **, * are significant at the 1%, 5%, and 10% levels, respectively. The *t*-value is in parentheses.

**Table 6:** The test of mediating mechanisms: Step 2.

	(1)	(2)	(3)	(4)
	MV = Internalizing Disorders	MV = Anxiety	MV = Neuroticism	MV = Withdrawal
	PYD_Toal	PYD_Toal	PYD_Toal	PYD_Toal
AQI_School	−0.014 ***	−0.014 ***	−0.014 ***	−0.014 ***
	(−4.76)	(−4.64)	(−4.44)	(−4.46)
MV	−0.027 ***	−0.073 ***	−0.099 ***	−0.153 ***
	(−38.77)	(−34.12)	(−24.20)	(−29.67)
Gender	0.01	0.028	−0.043 *	−0.044 **
	(0.49)	(1.32)	(−1.93)	(−2.05)
Growth_Place	0.016	0.021	0.02	0.015
	(0.81)	(1.03)	(0.90)	(0.69)
One_Child	−0.018	−0.025	−0.016	−0.006
	(−0.79)	(−1.08)	(−0.64)	(−0.26)
Local	0.027	0.031	0.04	0.041
	(1.04)	(1.15)	(1.41)	(1.48)
Father_Degree	0.022	0.025 *	0.041 ***	0.023
	(1.55)	(1.73)	(2.65)	(1.54)
Mother_Degree	0.051 ***	0.054 ***	0.059 ***	0.062 ***
	(3.50)	(3.58)	(3.71)	(4.00)
Family	−0.052	−0.079 *	−0.114 **	−0.096 **
	(−1.19)	(−1.77)	(−2.39)	(−2.09)
Income	0.012 **	0.014 **	0.013 **	0.012 **
	(2.26)	(2.53)	(2.22)	(2.06)
_cons	6.292 ***	6.111 ***	5.799 ***	5.992 ***
	(54.16)	(51.16)	(45.99)	(48.83)
*N*	3903	3903	3903	3903
adj. *R*^2^	0.296	0.249	0.152	0.205

Notes. ***, **, * are significant at the 1%, 5%, and 10% levels, respectively. The *t*-value is in parentheses.

## Data Availability

The data presented in this study are available on request from the corresponding author. The data are not publicly available due to being part of an ongoing project and privacy reasons.
